# Correlation Between Clinicopathological Findings, Cervical Smear, and HPV Results: A Retrospective Study of Women Referred for Colposcopy

**DOI:** 10.3390/diagnostics16213685

**Published:** 2026-11-07

**Authors:** Naile Kökbudak, Merve Solmaz, Fahriye Kılınç, Kübra Gündoğan, Zeliha Çelik, Pembe Oltulu

**Affiliations:** 1 Department of Medical Pathology, Faculty of Medicine, Necmettin Erbakan University, Hocacihan Neighborhood, Sultan Abdulhamid Han Street, No: 3, Selçuklu, 42080 Konya, Türkiye; merveozersolmaz@gmail.com (M.S.); drfahriyek@gmail.com (F.K.); zelis_ypr@hotmail.com (Z.Ç.); drpembe@yahoo.com (P.O.); 2 Department of Obstetrics and Gynecology, Faculty of Medicine, Necmettin Erbakan University, 42080 Konya, Türkiye

**Keywords:** cervical intraepithelial neoplasia, colposcopy, Papanicolaou test, cytology, HPV DNA test, HPV-16, HSIL, diagnostic accuracy, verification bias

## Abstract

**Background/Objectives:** This study evaluated the association of cervical cytology, human papillomavirus (HPV) genotypes, and colposcopic findings with histopathological outcomes in women referred for colposcopy. **Methods:** This retrospective cross-sectional study included 756 women who underwent colposcopy with cervical biopsy at a tertiary center between January 2022 and June 2025 and had HPV genotyping and conventional cytology results. The primary endpoint was cervical intraepithelial neoplasia grade 2 or worse (CIN2+) on biopsy; the highest grade across biopsy and conization specimens and CIN3+ were secondary endpoints. Associations were assessed using multivariable logistic regression, and diagnostic accuracy was reported according to STARD 2015. **Results:** CIN2+ was found in 85 women (11.2%). HPV-16 positivity (OR: 2.87; 95% CI: 1.72–4.77), epithelial cell abnormality on cytology (OR: 4.28; 95% CI: 2.05–8.92), and positive colposcopy (OR: 3.39; 95% CI: 2.00–5.73) were independently associated with higher odds of CIN2+. Adding colposcopy to a pre-colposcopy model increased the AUC from 0.730 to 0.770 (*p* = 0.034; optimism-corrected 0.753). Colposcopy had a sensitivity of 71.8% (95% CI: 61.4–80.2%) and an NPV of 94.6% (95% CI: 92.1–96.3%), but 24 of 85 CIN2+ cases (28.2%) had a negative colposcopic impression; results were similar for the highest-grade endpoint (92 CIN2+ cases). **Conclusions:** In this referral population, HPV-16, cytological abnormality, and colposcopic impression provided complementary information on the odds of CIN2+. Because more than one-quarter of CIN2+ cases were not colposcopically apparent, a negative colposcopy should not be considered sufficient to exclude high-grade disease, particularly in women with HPV-16 positivity or abnormal cytology. Prospective validation is required before these associations are used for triage.

## 1. Introduction

According to GLOBOCAN 2022 estimates, cervical cancer remains among the ten most common cancers and the ten leading causes of cancer death worldwide, with 661,021 new cases and 348,189 deaths in 2022 [1], and its burden remains highly unequal across countries, which underlies the World Health Organization (WHO) initiative to eliminate cervical cancer as a public health problem [2]. In Türkiye, the more recent GLOBOCAN 2024 estimates indicated 2577 new cases of cervical cancer and 1003 cervical cancer-related deaths in 2024, and the share of cervical cancer within the total cancer burden is lower than the global average [3].

Although the relatively low incidence and mortality ranking of cervical cancer in Türkiye reflects the contribution of screening programs, the disease remains a significant public health issue, and there is room to further improve the effectiveness of screening.

Persistent infection with high-risk HPV (hrHPV) is the necessary cause of virtually all cervical cancers, and cervical screening has therefore shifted from cytology alone towards HPV-based strategies [4,5]. In these strategies, HPV-positive women are triaged by HPV genotyping and/or cytology, and women whose risk is sufficiently high are referred for colposcopy and colposcopy-directed biopsy. Risk-based management, as formalized in the 2019 American Society for Colposcopy and Cervical Pathology (ASCCP) consensus guidelines, combines current test results and screening history to estimate the immediate risk of CIN3+ [6].

Nevertheless, risk stratification with currently available tools remains imperfect. Most HPV-positive women do not have precancer, cytology has limited sensitivity and reproducibility, and many assays report non-16/18 high-risk genotypes only as a pooled category, although the risk conferred by individual genotypes differs considerably. For this reason, extended genotyping and p16/Ki-67 dual-stain cytology have been incorporated into recent management recommendations [7,8], and molecular biomarkers, such as non-coding RNAs, are being investigated as future triage tools [9]. In routine practice, however, decisions at colposcopy are still largely based on HPV genotype, cytology, and the colposcopic impression. The extent to which these factors predict high-grade histopathology varies across populations and settings, colposcopic impression is known to miss a proportion of high-grade lesions, and accuracy estimates for colposcopy depend on how histological verification is obtained [10].

In this study, we aimed to evaluate the associations between HPV genotypes, cervical cytology, colposcopic findings, and histopathological outcomes in women referred for colposcopy at a tertiary center in Türkiye, to identify factors independently associated with CIN2+, and to estimate the diagnostic performance of colposcopic impression, taking into account the reference standard used and the potential for verification bias.

## 2. Materials and Methods

### 2.1. Study Design and Population

Women who underwent colposcopy at the outpatient clinics of the Department of Obstetrics and Gynecology, Necmettin Erbakan University Faculty of Medicine Hospital (Konya, Türkiye), between January 2022 and June 2025, and for whom colposcopy, HPV test, cervical smear, and cervical biopsy results were available, were included in this retrospective cross-sectional study. Indications for colposcopy included HPV positivity (HPV-16 and/or HPV-18, or other high-risk types), abnormal cervical cytology (ASC-US, ASC-H, AGC, LSIL, or HSIL), and clinically suspicious cervical lesions. The referral indication was recorded as stated in the referral and could be based on cytology, HPV, or histology results obtained before referral, often at other centers; the cytology analyzed in this study was the conventional smear evaluated in our laboratory.

Exclusion criteria were pregnancy, history of surgery for gynecologic malignancy, vaginal cuff cytology, missing smear, HPV, or pathology results, and prior treatment for cervical pre-invasive lesions. Of the 780 records retrieved, 24 were excluded: 10 women without a cervical biopsy (all of whom underwent direct excisional treatment), 1 with a nondiagnostic smear, 1 with an invalid HPV result, 3 with glandular lesions on biopsy (2 adenocarcinomas and 1 adenocarcinoma in situ), which were excluded because the analysis focused on HPV-associated squamous lesions, and 9 repeat records of women who were already included, so that each woman contributed only her first colposcopy episode. Thus, 756 women were included in the final analysis.

Approval for the study was obtained from the Non-Pharmaceutical and Non-Medical Device Research Ethics Committee of Necmettin Erbakan University (meeting No. 221, decision No. 2025/5835; dated 13 June 2025). The approved protocol defined the study period as January 2022 to June 2025. All data were extracted retrospectively from hospital archives after ethical approval had been obtained, and no records after June 2025 were included; all records concerned diagnostic procedures that had already been completed as part of routine clinical care, and no study-related intervention was performed. The study was conducted in accordance with the Declaration of Helsinki, and all data were anonymized prior to analysis.

### 2.2. Data Collection

Data collected for eligible patients included age, history of childbirth, mode of delivery, menopausal status, immunosuppression status, indication for and findings of colposcopy, HPV genotyping results (HPV-16, HPV-18, other high-risk types, and low-risk types), conventional smear results (based on the Bethesda classification), biopsy results (LSIL, HSIL, SCC, inflammation, metaplasia), the clock positions of the biopsies, and conization results.

### 2.3. Cervical Cytology and HPV Testing

**Figure 1:**
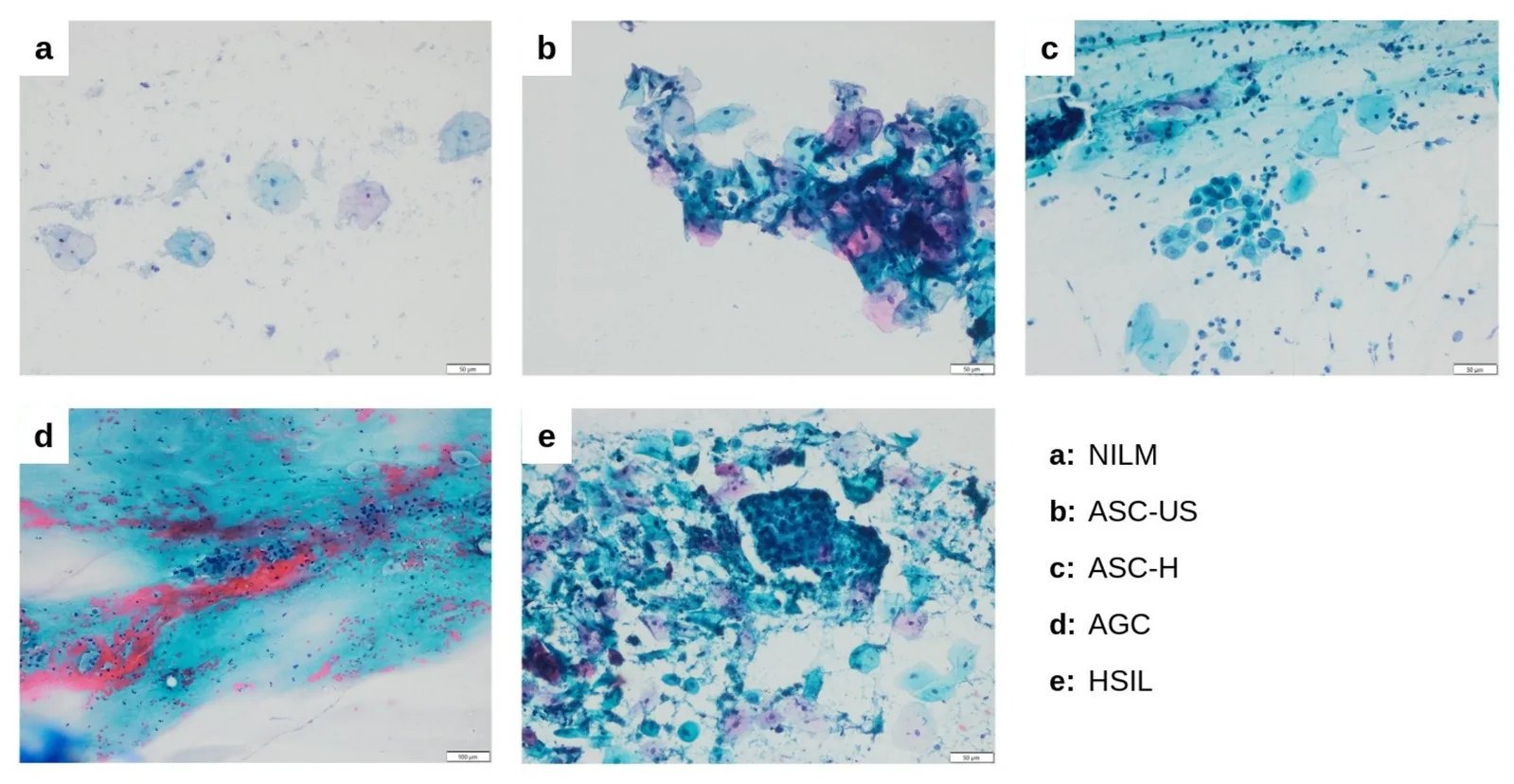
Representative conventional cervical smear findings (Papanicolaou stain): (**a**) negative for intraepithelial lesion or malignancy; (**b**) ASC-US; (**c**) ASC-H; (**d**) AGC; (**e**) HSIL. Scale bars: 50 µm (**a**–**c**,**e**) and 100 µm (**d**).

Cervical cytology samples were prepared using the conventional method, stained with the Papanicolaou technique, and reported according to the 2014 Bethesda System in the Pathology Laboratory of Necmettin Erbakan University Faculty of Medicine Hospital (Figure 1). Epithelial cell abnormality was defined as the presence of ASC-US, ASC-H, LSIL, HSIL, or atypical glandular cells (AGC; formerly AGUS). Inflammatory changes were recorded as mild inflammation, inflammation, or severe inflammation, and atrophic smears were recorded separately.

HPV DNA testing with genotyping was performed on cervical samples by multiplex real-time polymerase chain reaction (PCR) using the NeoPlex™ HPV29 Detection Kit (GeneMatrix Inc., Seongnam, Republic of Korea). Results were reported as HPV-16, HPV-18, other high-risk HPV types (31, 33, 35, 39, 45, 51, 52, 53, 56, 58, 59, 66, and 68), and low-risk HPV types; type-specific results for the non-16/18 high-risk genotypes were not available in the laboratory reports, and this pooled category also included HPV-53 and HPV-66, which have lower carcinogenic potential than the other types. Because co-infections were possible, a woman could be positive for more than one HPV category, and each category was analyzed as a separate binary variable. Positivity for two or more of the high-risk categories (HPV-16, HPV-18, and other high-risk HPV) was recorded as multiple high-risk infection; multiple infections within the pooled category could not be identified.

### 2.4. Colposcopy and Biopsy

Colposcopic examinations were performed by gynecologists of the Department of Obstetrics and Gynecology after the application of acetic acid, as part of routine care and with HPV and cytology results available to the colposcopist. Colposcopic findings were recorded as acetowhite epithelium, mosaicism, punctation, atypical vascularization, and border-in-border (inner border) sign, in line with the International Federation for Cervical Pathology and Colposcopy (IFCPC) terminology; the presence of any of these findings was considered a positive colposcopy.

Colposcopy-directed (targeted) biopsies were obtained from the most abnormal-appearing areas in women with visible lesions. In women without a visible colposcopic lesion, random biopsies were obtained from the transformation zone, predominantly at the 3, 6, 9, and 12 o’clock positions. HPV testing and cytology results were available at the time of colposcopy, biopsies were obtained during the index colposcopy, and conization was performed after the biopsy results were available, according to clinical indications; the exact intervals between these procedures, and any intervening treatment, were not systematically documented in the retrospective records. Endocervical curettage was not routinely performed, and the transformation zone type was not consistently documented in the retrospective records. The clock positions (1–12 o’clock) of all cervical biopsies were recorded, and the number of biopsies per woman was derived from the recorded positions. For quadrant-based analysis, clock positions were grouped as Q1 (1–3 o’clock), Q2 (4–6 o’clock), Q3 (7–9 o’clock), and Q4 (10–12 o’clock); in addition, the right (Q1+Q2), left (Q3+Q4), top (Q1+Q4), and bottom (Q2+Q3) halves of the cervix were evaluated.

### 2.5. Histopathological Evaluation

**Figure 2:**
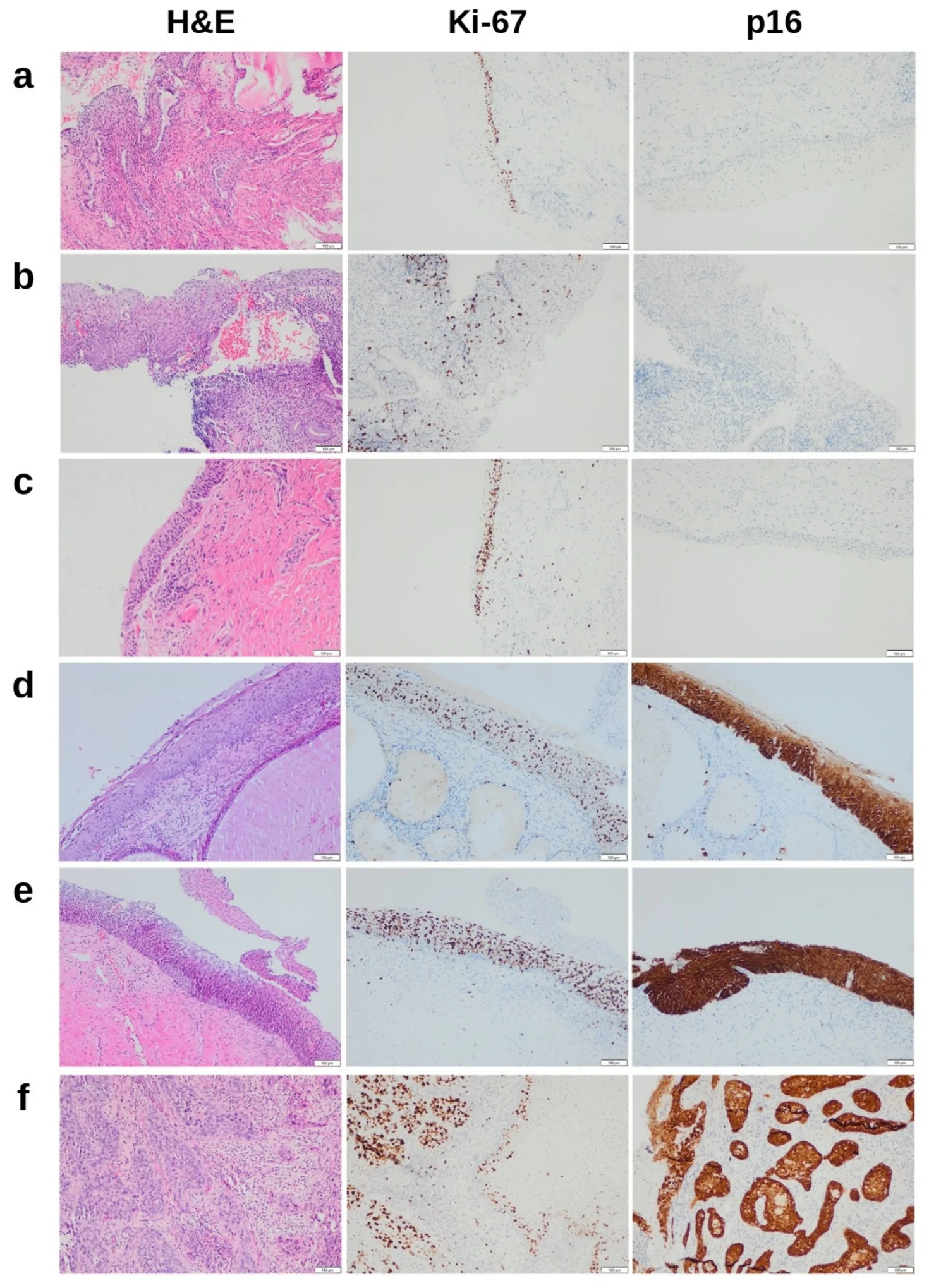
Representative histopathological findings of cervical biopsies with H&E (left column), Ki-67 (middle column), and p16 (right column) staining: (**a**) chronic cervicitis; (**b**) squamous metaplasia; (**c**) LSIL (CIN1); (**d**) HSIL (CIN2); (**e**) HSIL (CIN3); (**f**) squamous cell carcinoma. Note the basally restricted Ki-67 expression and negative p16 staining in (**a**–**c**) and the diffuse block-type p16 positivity with increased Ki-67 proliferation in (**d**–**f**). Scale bars: 100 µm.

Biopsy and conization specimens were fixed in 10% neutral buffered formalin, routinely processed, embedded in paraffin, and stained with hematoxylin and eosin (H&E). Squamous intraepithelial lesions were classified according to the two-tiered terminology as LSIL (CIN1) and HSIL (CIN2 or CIN3). In accordance with the Lower Anogenital Squamous Terminology (LAST) consensus recommendations, p16 immunohistochemistry was applied to morphologically equivocal lesions, particularly to distinguish HSIL (CIN2) from LSIL and from HSIL mimics such as immature squamous metaplasia and atrophy [11]; Ki-67 was used as an ancillary proliferation marker (Figure 2).

The primary endpoint was CIN2+ on cervical biopsy (HSIL/CIN2, HSIL/CIN3, or SCC); the comparison group (less than CIN2, <CIN2) included chronic cervicitis, squamous metaplasia, and LSIL/CIN1 (one verrucous squamous papilloma was classified with chronic cervicitis). Because 53 women also underwent conization, two secondary endpoints were defined: (i) CIN2+ according to the highest histological grade identified across the biopsy and conization specimens (highest-grade CIN2+) and (ii) CIN3+ (HSIL/CIN3 or SCC) on biopsy. Concordance between biopsy and conization diagnoses was assessed in women who underwent both procedures. Biopsy histopathology was used as the primary reference standard for the evaluation of colposcopy.

### 2.6. Statistical Analysis

Descriptive and comparative analyses were performed using IBM SPSS Statistics for Windows, version 22.0 (IBM Corp., Armonk, NY, USA), and the additional analyses performed during revision (ROC analysis, DeLong test, bootstrap validation, and false discovery rate correction) were performed using Python 3.13 (NumPy, SciPy, and pandas). Continuous variables were expressed as median (interquartile range, IQR), and categorical variables as number (percentage). The Pearson chi-square test or Fisher’s exact test (for 2 × 2 tables with any expected count < 5) was used to compare categorical variables. Normality of continuous variables was assessed using histograms, probability plots, and the Kolmogorov–Smirnov and Shapiro–Wilk tests, and the Mann–Whitney U test was used to compare two independent groups for non-normally distributed data. Multivariable logistic regression, including age, menopausal status, HPV genotype categories, epithelial cell abnormality on cytology, and colposcopic result, was performed to identify factors independently associated with CIN2+; the same model was fitted for the two secondary endpoints. Multicollinearity was assessed using variance inflation factors (VIFs). Because colposcopic impression guides biopsy site selection and is therefore not fully independent of the histological outcome, the odds ratio for colposcopy was interpreted as reflecting its added value over pre-colposcopy factors (age, HPV genotype, and cytology). To quantify this incremental value, a pre-colposcopy model (age, HPV genotype categories, and cytology) was compared with a model additionally including colposcopic impression; menopausal status was omitted from these two models because of its strong correlation with age. Discrimination was assessed using the area under the receiver operating characteristic curve (AUC) with 95% confidence intervals (CIs), and AUCs were compared using the DeLong test and a likelihood-ratio test. Calibration was assessed using the Hosmer–Lemeshow test, a calibration plot by deciles of predicted probability, and the calibration slope, and internal validation was performed using 500 bootstrap resamples drawn with replacement: in each resample, the model was refitted, and its AUC and calibration slope were evaluated both in the bootstrap sample and in the original sample; the mean difference between the two (optimism) was subtracted from the apparent performance to obtain optimism-corrected estimates. The calibration plot shows the apparent performance of the model in the study data. Results are presented as odds ratios (ORs) with 95% CIs, which describe associations with the odds of CIN2+ rather than relative risks. Agreement between colposcopy and biopsy results was assessed using Cohen’s kappa (κ).

The diagnostic performance of colposcopic impression was evaluated by calculating sensitivity, specificity, positive predictive value (PPV), negative predictive value (NPV), and likelihood ratios, with 95% CIs (Wilson score method for proportions and the log method for likelihood ratios), against biopsy CIN2+, highest-grade CIN2+, and CIN3+, and was reported in accordance with the Standards for Reporting of Diagnostic Accuracy Studies (STARD) 2015 [12] (Supplementary Table S4). The proportion of CIN2+ cases with a negative colposcopic impression (the proportion of disease missed by colposcopic impression) and the probability of CIN2+ among women with a negative colposcopy were reported separately. Analyses of biopsy localization and of HPV genotype subgroups were considered exploratory, and Benjamini–Hochberg false discovery rate (FDR)-adjusted q values were calculated within each of these tables to account for multiple comparisons. Statistical significance was set at *p* < 0.05.

## 3. Results

### 3.1. Study Population and Patient Characteristics

**Figure 3:**
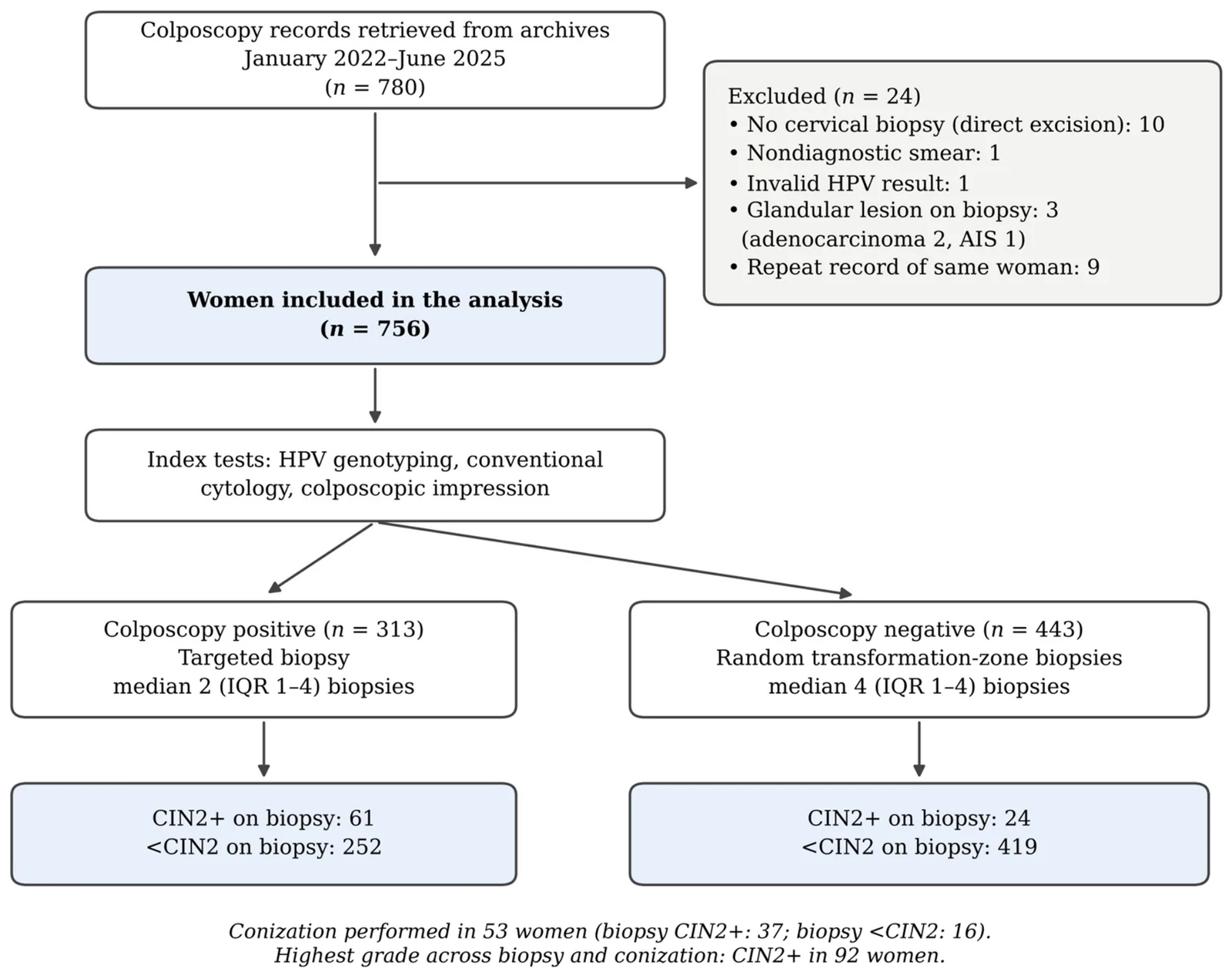
Flow diagram of the study population (STARD). AIS: adenocarcinoma in situ; IQR: interquartile range.

The selection of the study population is shown in Figure 3. Of 780 records, 24 were excluded, and 756 women were included. The median number of biopsies per woman was 3 (IQR 1–4): 4 (IQR 1–4) in women with a negative colposcopy, in whom random transformation-zone biopsies were taken, and 2 (IQR 1–4) in women with a positive colposcopy, in whom targeted biopsies were obtained; the biopsy clock positions were not recorded in 17 women.

The median age was 44 (37–51) years, and 38.2% (*n* = 289) were aged 40–49 years. Of the 749 women with available obstetric history, 91.9% (*n* = 688) had a history of childbirth, and 32.7% (*n* = 247) of all women were menopausal (Table 1).

Analysis of HPV type distribution showed HPV-16 in 24.1% (*n* = 182) of cases, HPV-18 in 11.6% (*n* = 88), other high-risk HPV types in 57.0% (*n* = 431), and low-risk HPV types in 21.7% (*n* = 164). In total, 180 women (23.8%) were negative for all high-risk HPV categories, and 107 (14.2%) were positive for two or more high-risk categories. An epithelial cell abnormality was detected in 6.9% (*n* = 52) of smear evaluations, with ASC-US (78.8%, *n* = 41) being the most common finding (Table 1).

Cervical biopsy results revealed chronic cervicitis in 70.6% (*n* = 534) of cases, squamous metaplasia in 11.5% (*n* = 87), LSIL (CIN1) in 6.6% (*n* = 50), HSIL (CIN2) in 4.2% (*n* = 32), HSIL (CIN3) in 6.0% (*n* = 45), and squamous cell carcinoma (SCC) in 1.1% (*n* = 8). The prevalence of CIN2+ on biopsy was 11.2% (*n* = 85), and that of CIN3+ was 7.0% (*n* = 53). Conization was performed in 53 women (7.0%), and HSIL (CIN3) was the most common conization result (39.6%, *n* = 21) (Table 1).

The most common indication for colposcopy was HPV positivity (81.2%, *n* = 614). Positive colposcopic findings were detected in 41.4% (*n* = 313) of cases, with acetowhite epithelium being the most frequent finding (71.6%, *n* = 224) (Table 2). The referral indication was HSIL in 11 women, whereas HSIL was identified in only 3 of the smears evaluated in our laboratory; this difference reflects that referral indications were based on cytology, HPV, or histology results obtained before referral, often at other centers, rather than on the smear analyzed in this study.

### 3.2. Comparison According to Biopsy CIN2+ Status

The median age of women with CIN2+ on biopsy (40 years) was significantly lower than that of women with <CIN2 (45 years) (*p* < 0.001), and the proportion of women younger than 40 years was higher in the CIN2+ group (43.5% vs. 31.1%; *p* = 0.006). The proportion of menopausal women was lower in the CIN2+ group than in the <CIN2 group (21.2% vs. 34.1%; *p* = 0.016) (Table 3).

HPV-16 positivity (49.4% vs. 20.9%; *p* < 0.001) and epithelial cell abnormality on the Pap smear (17.6% vs. 5.5%; *p* < 0.001) were significantly more frequent in the CIN2+ group, whereas negativity for all high-risk HPV categories was less frequent (10.6% vs. 25.5%; *p* = 0.002). No significant difference was observed between the groups regarding the presence of HPV-18, other high-risk HPV types, or low-risk HPV types (*p* > 0.05) (Table 3).

**Figure 4:**
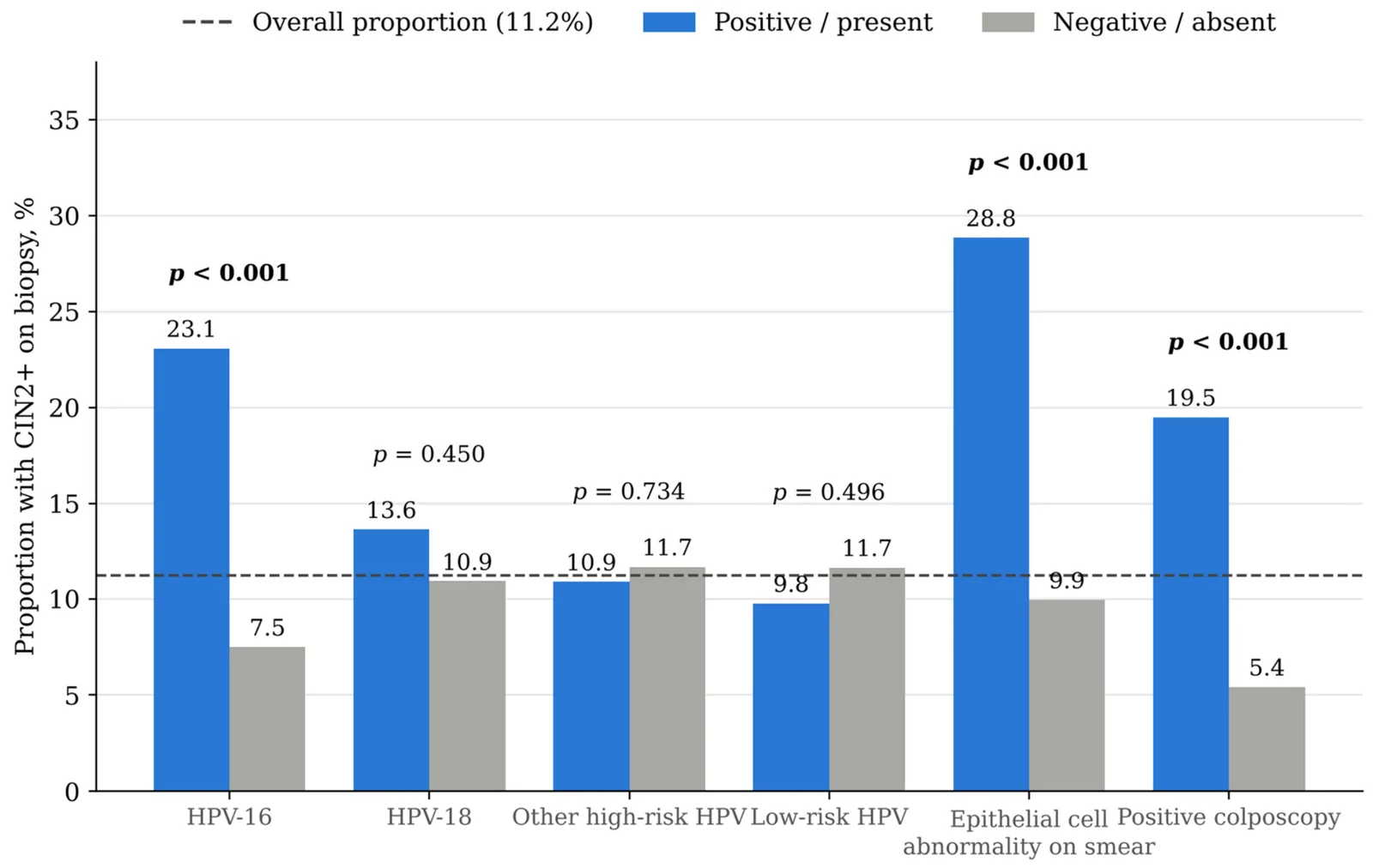
Proportion of CIN2+ (HSIL/CIN2, HSIL/CIN3, or SCC) on cervical biopsy according to the presence (blue) or absence (gray) of HPV genotypes, epithelial cell abnormality on cytology, and positive colposcopy. Values above the bars are percentages; the dashed line indicates the overall proportion in the cohort (11.2%; 85/756). *p* values were obtained using the chi-square test.

The proportion of CIN2+ according to HPV genotype, cytology, and colposcopy status is summarized in Figure 4: it reached 23.1% in HPV-16-positive women, 28.8% in women with an epithelial cell abnormality on cytology, and 19.5% in women with a positive colposcopy, compared with 11.2% in the whole cohort. When HPV-16 positivity and an epithelial cell abnormality were both present, 10 of 17 women (58.8%) had CIN2+, compared with 38 of 539 (7.1%) when both were absent.

Colposcopic positivity was significantly higher in the CIN2+ group (71.8% vs. 37.6%; *p* < 0.001). Among women with a positive colposcopy, mosaicism was observed more frequently in the CIN2+ group (21.3% [13/61] vs. 9.5% [24/252]; *p* = 0.011). Conization was performed in 43.5% of women in the CIN2+ group compared with 2.4% in the <CIN2 group (*p* < 0.001) (Table 3).

In the CIN2+ group, biopsies were less frequently obtained from the 3, 6, 9, and 12 o’clock positions than in the <CIN2 group. On a quadrant basis, biopsies from Q1, Q3, and Q4 and from the left, top, and bottom halves of the cervix were less frequent in the CIN2+ group, whereas no significant difference was found for Q2 (*p* = 0.074) or the right half (*p* = 0.120); all of these differences remained significant after FDR correction (Table 4). Because random biopsies were taken from standard positions in colposcopy-negative women, these differences reflect the biopsy protocol rather than the anatomical distribution of disease.

### 3.3. Factors Associated with CIN2+, Incremental Value of Colposcopy, and Diagnostic Performance

**Figure 5:**
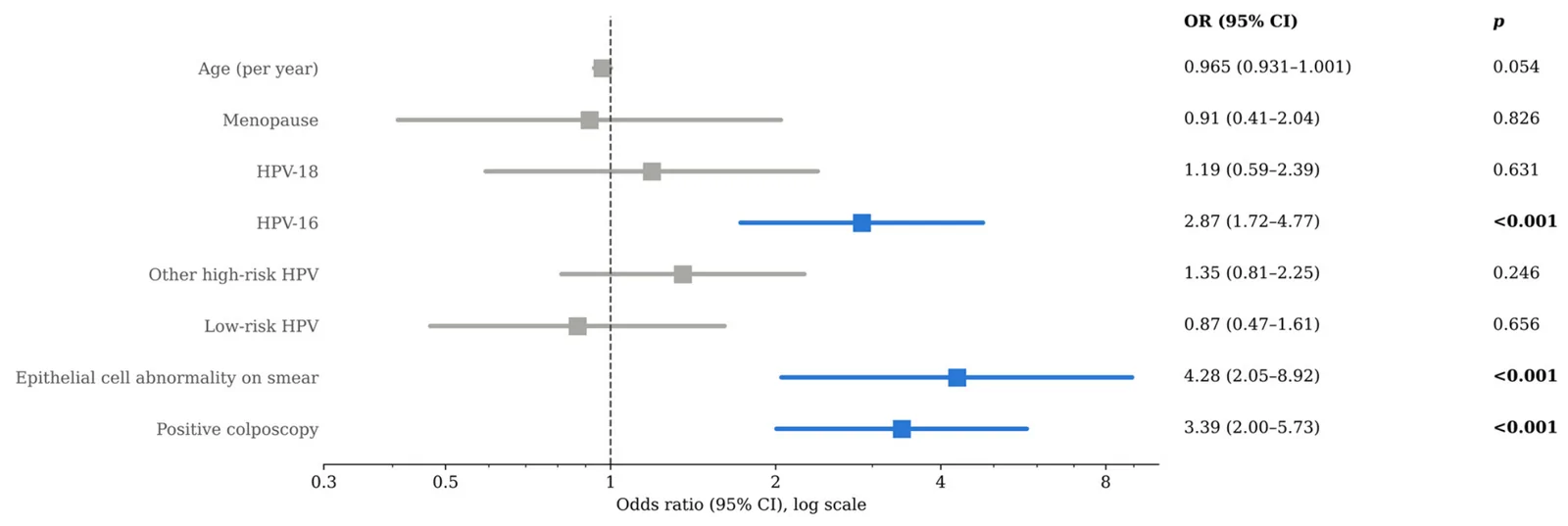
Forest plot of the multivariable logistic regression analysis showing factors independently associated with CIN2+ on cervical biopsy. Squares indicate odds ratios and horizontal lines indicate 95% confidence intervals (log scale); significant variables are shown in blue. The dashed vertical line indicates OR = 1.

In multivariable logistic regression, HPV-16 positivity (OR: 2.87; 95% CI: 1.72–4.77; *p* < 0.001), an epithelial cell abnormality on the Pap smear (OR: 4.28; 95% CI: 2.05–8.92; *p* < 0.001), and a positive colposcopy (OR: 3.39; 95% CI: 2.00–5.73; *p* < 0.001) were independently associated with higher odds of CIN2+. Older age showed a borderline inverse association (OR per year: 0.965; 95% CI: 0.931–1.001; *p* = 0.054). Age and menopausal status were correlated (r = 0.73), although the VIFs were acceptable (2.1 for both; ≤1.1 for all other variables), and age was significantly associated with lower odds of CIN2+ when menopausal status was omitted (OR: 0.963; 95% CI: 0.938–0.988; *p* = 0.004). The model showed adequate calibration (Hosmer–Lemeshow χ^2^ = 8.84; *p* = 0.356). The same three factors were independently associated with highest-grade CIN2+ and with CIN3+ (Table 5, Figure 5).

**Figure 6:**
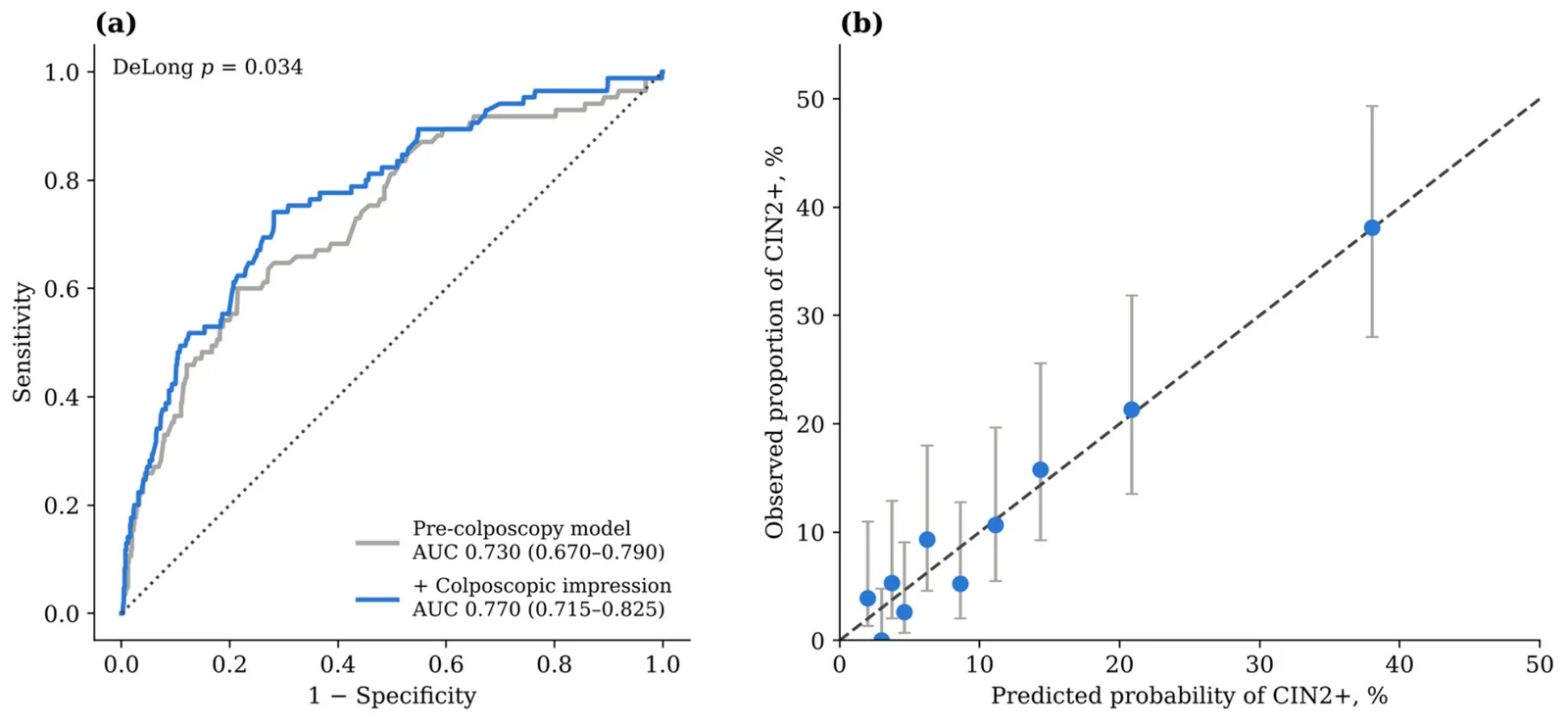
(**a**) Receiver operating characteristic curves of the pre-colposcopy model (age, HPV genotype categories, and cytology; gray) and of the model additionally including colposcopic impression (blue) for CIN2+ on biopsy, with AUCs and 95% confidence intervals; the dotted diagonal line indicates no discrimination (AUC = 0.5). (**b**) Calibration of the model including colposcopic impression: observed proportion of CIN2+ (with 95% Wilson confidence intervals) versus mean predicted probability in deciles of predicted probability; the dashed line indicates perfect calibration. The calibration plot shows apparent (not optimism-corrected) performance; optimism-corrected estimates are reported in the text.

The pre-colposcopy model including age, HPV genotype categories, and cytology had an AUC of 0.730 (95% CI: 0.670–0.790). Adding colposcopic impression increased the AUC to 0.770 (95% CI: 0.715–0.825; DeLong *p* = 0.034; likelihood-ratio χ^2^ = 22.4, *p* < 0.001) (Figure 6a). After bootstrap internal validation, the optimism-corrected AUCs were 0.714 and 0.753, respectively, and the optimism-corrected calibration slope of the full model was 0.92; observed and predicted proportions of CIN2+ agreed across deciles of predicted probability (apparent calibration; Figure 6b). Similar results were obtained for the highest-grade endpoint (AUC 0.725 vs. 0.761; DeLong *p* = 0.036).

Colposcopic impression showed slight agreement with biopsy histology (κ = 0.158; 95% CI: 0.078–0.237) (Table 6). Against biopsy CIN2+, colposcopy had a sensitivity of 71.8% (95% CI: 61.4–80.2%), a specificity of 62.4% (95% CI: 58.7–66.0%), a PPV of 19.5% (95% CI: 15.5–24.2%), and an NPV of 94.6% (95% CI: 92.1–96.3%) (Table 7). Importantly, 24 of the 85 CIN2+ cases (28.2%; 95% CI: 19.8–38.6%), including 13 HSIL (CIN3) lesions and 1 SCC, had a negative colposcopic impression and were detected only by random biopsies. Thus, although the probability of CIN2+ after a negative colposcopy was 5.4% (24/443), more than one-quarter of the high-grade lesions in this cohort were not colposcopically apparent. Among colposcopy-negative women, CIN2+ was found in 12.7% (9/71) of HPV-16-positive women and in 12.9% (4/31) of women with an epithelial cell abnormality, compared with 3.8% (13/346) of women with neither finding.

Among the 53 women who underwent both biopsy and conization, the CIN2+/<CIN2 classification was concordant in 35 (66.0%) (Table 8). Seven of the 16 women (43.8%) with <CIN2 on biopsy had CIN2+ on the conization specimen (four HSIL/CIN3 and three HSIL/CIN2). In 10 of these 16 women, the colposcopic impression was positive, and conization was performed because of discordance between the colposcopic impression and the biopsy result; for the remaining 6 women, who had a negative colposcopy, the specific indication was not consistently documented in the records; of these 6 women, 2 were HPV-16-positive, 2 were positive for other high-risk HPV types only (1 of whom was postmenopausal), and 2 were negative for high-risk HPV and had been referred because of a clinically suspicious cervical lesion, and conization revealed CIN2+ in 3 of them. Of all 16 women, 9 were HPV-16- and/or HPV-18-positive, and 7 were postmenopausal. Conversely, 11 of the 37 women (29.7%) with CIN2+ on biopsy had <CIN2 on conization. When the highest grade across biopsy and conization specimens was used as the reference standard, the number of CIN2+ cases increased from 85 to 92 (12.2%). With this endpoint, the sensitivity, specificity, PPV, and NPV of colposcopy were 70.7%, 62.7%, 20.8%, and 93.9%, respectively, and 27 of the 92 CIN2+ cases (29.3%) had a negative colposcopy (Table 7); the factors associated with CIN2+ were unchanged (Table 5).

### 3.4. Associations of HPV Genotypes

HPV-16-positive women less frequently had a history of childbirth (87.2% vs. 93.3%; *p* = 0.009). HPV-18 positivity was associated with the mode of delivery (*p* = 0.034), and immunosuppression was less frequent in women positive for other high-risk HPV types (3.5% vs. 7.1%; *p* = 0.027) (Supplementary Table S1).

HPV-16 positivity was associated with CIN2+ on biopsy (23.1% vs. 7.5%; *p* < 0.001), the distribution of biopsy diagnoses (*p* < 0.001), conization (14.3% vs. 4.7%; *p* < 0.001), and colposcopy positivity (61.0% vs. 35.2%; *p* < 0.001). HPV-18 positivity was associated with colposcopy positivity (58.0% vs. 39.2%; *p* < 0.001) and with the distribution of conization diagnoses (*p* = 0.012), although the latter comparison was based on only six HPV-18-positive women who underwent conization. Epithelial cell abnormality on cytology was less frequent in women positive for other high-risk HPV types (3.9% vs. 10.8%; *p* < 0.001) (Supplementary Table S2).

Regarding biopsy localization, biopsies from the 3, 9, and 12 o’clock positions were less frequent in HPV-16-positive women, and several position-specific differences were observed for HPV-18, other high-risk, and low-risk HPV types; however, none of the genotype-specific localization differences remained significant after FDR correction (all q > 0.15) (Supplementary Table S3).

## 4. Discussion

In this referral cohort, HPV-16 positivity was associated with approximately threefold higher odds of CIN2+ after adjustment for age, the other HPV categories, cytology, and colposcopic impression, and this association was consistent for the highest-grade and CIN3+ endpoints. HPV-16-positive women also more frequently underwent conization (14.3% vs. 4.7%). These findings are in line with the established hierarchy of genotype-specific risk. A systematic review of HPV genotyping in cervical cancer screening concluded that the separate identification of HPV-16, and to a lesser extent HPV-18, provides clinically meaningful risk stratification beyond pooled hrHPV results [13]; and a study from a colposcopy center in Türkiye reported that HPV-16 was the genotype most strongly associated with high-grade histology [14]. Nevertheless, genotyping alone is not sufficient for triage: among hrHPV-positive women with LSIL cytology, a triage model based on HPV-16/18 alone had a sensitivity of 54.9% and a specificity of 79.6% for CIN2+ [15]. Similarly, in our cohort, HPV-16 was present in 49.4% (42/85) of women with CIN2+, with a specificity of 79.1% (531/671). The CIN3+ proportion was 16.5% (30/182) in HPV-16-positive women and 4.0% (23/574) in HPV-16-negative women, which is relevant to the 2019 ASCCP guidelines, which recommend colposcopy when the immediate CIN3+ risk is 4% or higher and identify HPV-16 positivity as conferring the highest risk [6]. Of note, 9 of the 85 CIN2+ cases (10.6%) occurred in women who were negative for all high-risk HPV categories in our assay, which may reflect HPV-negative lesions, sampling or analytical limitations, or misclassification inherent to retrospective data.

HPV-18 and the pooled category of other high-risk types were not independently associated with CIN2+ in our analysis. This should not be interpreted as evidence that these genotypes lack clinical relevance. HPV-18 is preferentially associated with glandular and endocervical lesions, which are less accessible to colposcopy-directed biopsy and were excluded from our squamous-focused analysis; moreover, the number of HPV-18-positive women was modest, and the confidence interval of its OR (0.59–2.39) was wide. The pooled “other high-risk” category combines genotypes with very different carcinogenic potential, ranging from HPV-31, -33, -45, -52, and -58 to HPV-53, -56, -59, and -66, so that the higher risk associated with some types is diluted by the lower risk of others. Studies using extended genotyping have shown that types such as HPV-58, -52, -33, and -31 are frequent among CIN2+ lesions and can refine triage beyond HPV-16/18 [15], a risk-based strategy using genotype-specific immediate risks achieved higher specificity and required fewer colposcopies than conventional HPV-based screening [16], and recent enduring guidelines now recommend genotype-specific management of positive HPV results according to a risk hierarchy (16, 18, 45, 33, 31, 52, 58, 35, 39, 51, 59, 56, 68, and 66) [7]. Because type-specific results were not available in our archival reports and multiple infections within the pooled category could not be distinguished, our findings for the non-16 genotypes should be interpreted with caution.

The presence of an epithelial cell abnormality on the smear was associated with approximately fourfold higher odds of identifying CIN2+ on cervical biopsy. In line with this, Ittiamornlert et al. showed that among women with ASC-US cytology, hrHPV genotyping helps identify those harboring underlying high-grade lesions, emphasizing that even minor cytological abnormalities carry a meaningful risk when combined with HPV status [17]. Moreover, in a large cohort of women with histologic LSIL followed for at least two years, a preceding cytology of ASC-H or worse and HPV-16 infection were associated with lesion progression, indicating that the cytological grade carries prognostic information beyond the initial biopsy result [18]. The overall prevalence of epithelial cell abnormalities (6.9%) was low; this is explained by the predominantly HPV-driven referral pathway, in which 81.2% of women were referred because of a positive HPV test and most had negative cytology. In this setting, cytology acted as a triage test with low sensitivity (17.6%) but high specificity (94.5%) for CIN2+, and an abnormal result substantially increased the probability of CIN2+ (28.8%). When HPV-16 positivity and an epithelial cell abnormality were both present, more than half of the women (58.8%) had CIN2+, compared with 7.1% when both were absent. These observations support the combined use of genotype and cytology information, as in the current risk-based framework [6].

A positive colposcopic impression was associated with approximately threefold higher odds of CIN2+, and adding colposcopy to a pre-colposcopy model modestly improved discrimination (AUC from 0.730 to 0.770; optimism-corrected, from 0.714 to 0.753). This incremental value must be interpreted cautiously, because colposcopic impression determines where targeted biopsies are taken and is therefore not fully independent of the histological outcome. Overall, colposcopy showed only slight agreement with histology (κ = 0.158), with a sensitivity of 71.8% and a specificity of 62.4%, consistent with studies reporting that the accuracy of colposcopic impression is imperfect and varies according to referral cytology, HPV status, and transformation zone type [10,19]. The most clinically relevant finding is that 24 of the 85 CIN2+ cases (28.2%), including 13 CIN3 lesions and 1 SCC, had a negative colposcopic impression and were detected only through random transformation-zone biopsies. Two measures that are often conflated should be distinguished: the probability of CIN2+ among colposcopy-negative women (5.4%, i.e., 1 − NPV), which depends on the prevalence of disease in the referral population, and the proportion of CIN2+ cases missed by colposcopic impression (28.2%, i.e., 1 − sensitivity). Accordingly, the NPV of 94.6% does not indicate that a negative colposcopy reliably excludes high-grade disease. In our data, the probability of CIN2+ after a negative colposcopy was 12.7% in HPV-16-positive women and 12.9% in women with abnormal cytology, compared with 3.8% in women with neither finding, which indicates that the clinical implications of a negative colposcopy depend on the pre-colposcopy risk. These findings support obtaining random biopsies when no lesion is visible, particularly in women at higher pre-colposcopy risk; in a prospective study of women referred with low-grade cytology and a normal colposcopy, taking four random biopsies doubled the detection of CIN2+ compared with a single biopsy [20].

The reliability of the reference standard directly influences the estimates of diagnostic accuracy. In women who underwent both procedures, the biopsy and conization classifications were discordant in one-third of cases, and 7 of the 16 women with <CIN2 on biopsy had CIN2+ on the excisional specimen, indicating that biopsy alone underestimates the prevalence of high-grade disease. Conversely, 11 of the 37 women with CIN2+ on biopsy had <CIN2 on conization, which may reflect the removal of small lesions by biopsy, regression, or sampling. Discordance between colposcopic biopsy and loop excision has also been reported in a Turkish series [21]. When the highest grade across both specimens was used, the number of CIN2+ cases increased from 85 to 92, but the estimates of colposcopic performance and the factors associated with CIN2+ were essentially unchanged. However, conization was performed selectively, and the histological verification of colposcopy-negative women relied on random biopsies without routine endocervical curettage. In a large series of 47,134 women who underwent colposcopy-directed ectocervical biopsy and endocervical curettage, HSIL or worse was diagnosed in 14.4%, and endocervical curettage detected additional lesions, particularly in women aged 45 years or older, those with hrHPV infection or high-grade cytology, and those with a type 3 transformation zone [22]. Therefore, some high-grade lesions in colposcopy-negative women, especially small or endocervical lesions, may have been missed in our cohort; such partial and differential verification may have inflated the sensitivity and NPV of colposcopy.

CIN2+ was more frequent in younger women, and age showed a borderline inverse association with CIN2+ in the multivariable model, which became significant when menopausal status, with which age was strongly correlated, was omitted. This pattern may reflect the higher rate of persistent HPV infections leading to lesion formation in younger women. In addition, the retraction of the transformation zone into the endocervical canal after menopause limits colposcopic visualization and sampling in older women [22], so the lower proportion of CIN2+ in older women may partly be a detection artifact. Among colposcopic patterns, mosaicism was more frequent in CIN2+ lesions, consistent with the abnormal vascular architecture of neoplastic epithelium within the transformation zone, where HPV-associated HSIL has been shown to arise from reserve cells involved in squamous metaplasia [23]; however, this comparison was restricted to colposcopy-positive women and was based on small numbers. Regarding biopsy localization, biopsies in the CIN2+ group, as well as in HPV-16-positive women, were less frequently obtained from the 3, 6, 9, and 12 o’clock positions. This difference most likely reflects the biopsy protocol rather than a biological predilection of lesions for particular sites: in women without a visible lesion, random biopsies were taken predominantly from these standard positions, whereas in women with visible lesions, biopsies were directed to the abnormal area. The anatomical distribution of biopsies therefore cannot be interpreted as the anatomical distribution of disease, and the genotype-specific localization differences, which did not remain significant after correction for multiple testing, should be regarded as hypothesis-generating only.

Taken together, our results indicate that HPV-16 genotyping, cytology, and colposcopic impression provide complementary information on the probability of CIN2+ in women referred for colposcopy, but none of them is sufficient on its own, and their combination did not identify a group with negligible risk. These associations do not by themselves establish a new triage or management algorithm. Additional biomarkers, such as p16/Ki-67 dual-stain cytology, which has been incorporated into recent management recommendations for HPV-positive individuals [8], and emerging molecular markers, such as non-coding RNAs [9], may help refine risk stratification in the future; these were not evaluated in the present study and require prospective validation.

### Strengths and Limitations

The strengths of our study include a relatively large referral cohort, the simultaneous evaluation of HPV genotypes, cytology, colposcopy, and histopathology in the same women, the availability of conization results for a subset of women, and the reporting of diagnostic accuracy according to STARD with confidence intervals and internal validation. However, several limitations should be considered. First, the retrospective design relied on routinely collected clinical and pathology data that were not originally recorded for research purposes; therefore, misclassification and information bias cannot be ruled out, and data on smoking, HPV vaccination history, prior HPV persistence, and screening history were not available, which limits adjustment for confounding. Second, type-specific results for the non-16/18 high-risk genotypes were not available because these types were reported as a pooled category, so genotype-specific risks and multiple infections within this category could not be assessed; in addition, HPV testing was performed with a single multiplex real-time PCR assay, and the analytical performance and correlation with histology differ among commercial HPV assays.

Third, the study population consisted exclusively of women referred for colposcopy at a tertiary center, most of whom were referred because of a positive HPV test; this selected population differs from the general screening population, and predictive values, which depend on disease prevalence, cannot be directly transferred to other settings. In addition, ten women who underwent direct excisional treatment without a prior biopsy were excluded, which may have removed some women with a high pre-test probability of disease. Fourth, the reference standard was imperfect: biopsy sampling differed according to the colposcopic impression (targeted vs. random), endocervical curettage was not routinely performed, the transformation zone type was not consistently documented, and conization was performed selectively; moreover, the intervals between HPV testing, cytology, colposcopy, and conization, and any intervening treatment, were not systematically documented. Partial and differential verification bias may therefore have inflated the sensitivity and NPV of colposcopy, and endocervical lesions may have been missed, particularly in older and postmenopausal women. Colposcopists were not blinded to HPV and cytology results, as in routine practice, and p16 immunohistochemistry was applied selectively to equivocal lesions rather than to all biopsies, so interobserver variability in the distinction between CIN1 and CIN2 cannot be fully excluded.

Finally, the multivariable model was validated only internally, the analyses of biopsy localization and HPV genotype subgroups were exploratory, and the single-center design limits generalizability. Prospective multicenter studies with complete histological verification are needed to validate these findings.

## 5. Conclusions

In this single-center referral population, HPV-16 positivity, an epithelial cell abnormality on cytology, and a positive colposcopic impression were independently associated with higher odds of CIN2+, whereas no independent association was observed for HPV-18 or for the pooled category of other high-risk HPV types; the latter finding should be interpreted in light of the limited number of HPV-18-positive women and the pooled reporting of non-16/18 genotypes.

Colposcopic impression showed only slight agreement with histology. Although the probability of CIN2+ after a negative colposcopy was low overall (5.4%), more than one-quarter of CIN2+ cases had a negative colposcopic impression, and this probability was higher in women with HPV-16 positivity or abnormal cytology. Therefore, a negative colposcopy should not be regarded as sufficient to exclude high-grade disease, random biopsies remain important when no lesion is visible, and risk-based follow-up is required.

These findings support considering HPV-16 status and cytology together with colposcopic findings when interpreting colposcopy results in referral settings. However, because they are based on retrospective associations in a selected tertiary referral population, they do not establish a new triage strategy; prospective multicenter studies are needed to determine whether incorporating these factors into risk-based colposcopy management improves clinical outcomes and resource use.

## Figures and Tables

**Table 1:** Women’s age and certain reproductive characteristics, along with HPV types, smear cytology, biopsy, and conization results.

	*n* = 756
Age (years)	44 (37–51)
Age group	
<40	246 (32.5)
40–49	289 (38.2)
≥50	221 (29.2)
Childbirth status (*n* = 749)	688 (91.9)
Mode of delivery (*n* = 688)	
Vaginal delivery	376 (54.7)
Cesarean section	229 (33.3)
Both vaginal delivery and cesarean section	83 (12.1)
Menopause	247 (32.7)
Immunosuppression (*n* = 753)	38 (5.0)
HPV-16	182 (24.1)
HPV-18	88 (11.6)
Other high-risk HPV	431 (57.0)
Low-risk HPV	164 (21.7)
Negative for all high-risk HPV categories	180 (23.8)
≥2 high-risk HPV categories	107 (14.2)
Epithelial cell abnormality on smear	52 (6.9)
Type of epithelial cell abnormality (*n* = 52)	
ASC-US	41 (78.8)
ASC-H	4 (7.7)
AGC	3 (5.8)
LSIL	1 (1.9)
HSIL	3 (5.8)
Inflammation on smear	608 (80.4)
Inflammation findings (*n* = 608)	
Inflammation	311 (51.2)
Severe inflammation	245 (40.3)
Mild inflammation	52 (8.6)
Atrophy on smear	68 (9.0)
Cervical biopsy	
Chronic cervicitis ^a^	534 (70.6)
Squamous metaplasia	87 (11.5)
LSIL (CIN1)	50 (6.6)
HSIL (CIN2)	32 (4.2)
HSIL (CIN3)	45 (6.0)
SCC	8 (1.1)
Biopsy category	
CIN2+	85 (11.2)
<CIN2	671 (88.8)
CIN3+ on biopsy	53 (7.0)
Conization	53 (7.0)
Conization findings (*n* = 53)	
HSIL (CIN3)	21 (39.6)
HSIL (CIN2)	11 (20.8)
LSIL (CIN1)	9 (17.0)
Chronic cervicitis	6 (11.3)
Squamous metaplasia	5 (9.4)
SCC	1 (1.9)

Continuous variables are presented as median (first quartile–third quartile), and categorical variables as number (percentage). Epithelial cell abnormality: ASC-US or worse, including AGC. High-risk HPV categories: HPV-16, HPV-18, and other high-risk HPV. ^a^ Includes one verrucous squamous papilloma.

**Table 2:** Indications for colposcopy and colposcopy results.

	*n* = 756
Indication for colposcopy	
HPV	614 (81.2)
Cervical lesion	84 (11.1)
Direct smear result (ASC-US, ASC-H)	18 (2.4)
HSIL	11 (1.5)
LSIL	10 (1.3)
Other	19 (2.5)
Positive colposcopy	313 (41.4)
Colposcopy findings (*n* = 313)	
Acetowhite epithelium	224 (71.6)
Atypical vascularization	73 (23.3)
Mosaicism	37 (11.8)
Punctation	28 (8.9)
Border-in-border	3 (1.0)

Variables are presented as number (percentage). Referral indications were based on results obtained before referral and do not necessarily correspond to the smear evaluated in this study (Table 1). Colposcopic findings are not mutually exclusive.

**Table 3:** Distribution of women’s age, certain reproductive characteristics, HPV types, smear cytology, conization, and colposcopy results according to biopsy CIN2+ status.

	<CIN2 (*n* = 671)	CIN2+ (*n* = 85)	*p*
Age (years)	45 (38–52)	40 (35–46)	<0.001 *
Age group			0.006 *
<40	209 (31.1)	37 (43.5)	
40–49	254 (37.9)	35 (41.2)	
≥50	208 (31.0)	13 (15.3)	
Childbirth status	612 (92.2)	76 (89.4)	0.382
Mode of delivery			0.397
Vaginal delivery	340 (55.6)	36 (47.4)	
Cesarean section	200 (32.7)	29 (38.2)	
Both vaginal delivery and cesarean section	72 (11.8)	11 (14.5)	
Menopause	229 (34.1)	18 (21.2)	0.016 *
Immunosuppression	34 (5.1)	4 (4.7)	1.000
HPV-18	76 (11.3)	12 (14.1)	0.450
HPV-16	140 (20.9)	42 (49.4)	<0.001 *
Other high-risk HPV	384 (57.2)	47 (55.3)	0.734
Low-risk HPV	148 (22.1)	16 (18.8)	0.496
Negative for all high-risk HPV categories	171 (25.5)	9 (10.6)	0.002 *
Epithelial cell abnormality on smear	37 (5.5)	15 (17.6)	<0.001 *
Type of epithelial cell abnormality			0.134
ASC-US	31 (83.8)	10 (66.7)	
ASC-H	2 (5.4)	2 (13.3)	
AGC	3 (8.1)	0	
LSIL	0	1 (6.7)	
HSIL	1 (2.7)	2 (13.3)	
Inflammation on smear	542 (80.8)	66 (77.6)	0.494
Inflammation findings			0.428
Inflammation	276 (50.9)	35 (53.0)	
Severe inflammation	222 (41.0)	23 (34.8)	
Mild inflammation	44 (8.1)	8 (12.1)	
Atrophy on smear	60 (8.9)	8 (9.4)	0.887
Conization	16 (2.4)	37 (43.5)	<0.001 *
Conization findings			0.560
HSIL (CIN3)	4 (25.0)	17 (45.9)	
HSIL (CIN2)	3 (18.8)	8 (21.6)	
LSIL (CIN1)	4 (25.0)	5 (13.5)	
Chronic cervicitis	3 (18.8)	3 (8.1)	
Squamous metaplasia	2 (12.5)	3 (8.1)	
SCC	0	1 (2.7)	
Indication for colposcopy			<0.001 *
HPV	545 (81.2)	69 (81.2)	
Cervical lesion	80 (11.9)	4 (4.7)	
Direct smear result (ASC-US, ASC-H)	18 (2.7)	0	
HSIL	1 (0.1)	10 (11.8)	
LSIL	10 (1.5)	0	
Other	17 (2.5)	2 (2.4)	
Positive colposcopy	252 (37.6)	61 (71.8)	<0.001 *
Colposcopy findings (*n* = 313)			
Acetowhite epithelium	181 (71.8)	43 (70.5)	0.836
Atypical vascularization	56 (22.2)	17 (27.9)	0.349
Mosaicism	24 (9.5)	13 (21.3)	0.011 *
Punctation	19 (7.5)	9 (14.8)	0.076
Border-in-border	3 (1.2)	0 (0.0)	1.000

Continuous variables are presented as median (first quartile–third quartile), and categorical variables as number (percentage); * *p* < 0.05. Percentages of colposcopic findings are calculated among women with a positive colposcopy.

**Table 4:** Distribution of biopsy localization according to biopsy CIN2+ status.

	<CIN2 (*n* = 671)	CIN2+ (*n* = 85)	*p*
Biopsy localization			
1 o’clock position	54 (8.0)	11 (12.9)	0.129
2 o’clock position	23 (3.4)	1 (1.2)	0.506
3 o’clock position	364 (54.2)	22 (25.9)	<0.001 *
4 o’clock position	27 (4.0)	4 (4.7)	0.770
5 o’clock position	50 (7.5)	7 (8.2)	0.797
6 o’clock position	397 (59.2)	39 (45.9)	0.020 *
7 o’clock position	60 (8.9)	5 (5.9)	0.343
8 o’clock position	28 (4.2)	5 (5.9)	0.405
9 o’clock position	365 (54.4)	20 (23.5)	<0.001 *
10 o’clock position	25 (3.7)	4 (4.7)	0.557
11 o’clock position	59 (8.8)	7 (8.2)	0.864
12 o’clock position	396 (59.0)	29 (34.1)	<0.001 *
Quadrant localization			
Q1	429 (63.9)	31 (36.5)	<0.001 *
Q2	452 (67.4)	49 (57.6)	0.074
Q3	435 (64.8)	28 (32.9)	<0.001 *
Q4	464 (69.2)	39 (45.9)	<0.001 *
Right (Q1+Q2)	545 (81.2)	63 (74.1)	0.120
Left (Q3+Q4)	558 (83.2)	51 (60.0)	<0.001 *
Top (Q1+Q4)	544 (81.1)	56 (65.9)	0.001 *
Bottom (Q2+Q3)	546 (81.4)	59 (69.4)	0.009 *

Variables are presented as number (column percentage). Q1: 1–3 o’clock; Q2: 4–6 o’clock; Q3: 7–9 o’clock; Q4: 10–12 o’clock; * *p* < 0.05. All differences with *p* < 0.05 remained significant after Benjamini–Hochberg FDR correction (q < 0.05). Biopsy clock positions were not recorded in 17 women.

**Table 5:** Factors independently associated with CIN2+ on biopsy and with the secondary endpoints (multivariable logistic regression).

	Biopsy CIN2+ (*n* = 85)		Highest-Grade CIN2+ (*n* = 92)		Biopsy CIN3+ (*n* = 53)	
	OR (95% CI)	*p*	OR (95% CI)	*p*	OR (95% CI)	*p*
Age (per year)	0.965 (0.931–1.001)	0.054	0.967 (0.934–1.001)	0.058	0.976 (0.934–1.020)	0.279
Menopause	0.91 (0.41–2.04)	0.826	1.00 (0.46–2.15)	0.996	1.10 (0.42–2.88)	0.848
HPV-18	1.19 (0.59–2.39)	0.631	1.33 (0.69–2.57)	0.400	0.70 (0.26–1.89)	0.488
HPV-16	2.87 (1.72–4.77)	<0.001 *	3.04 (1.86–4.96)	<0.001 *	3.51 (1.88–6.56)	<0.001 *
Other high-risk HPV	1.35 (0.81–2.25)	0.246	1.28 (0.78–2.09)	0.324	1.15 (0.61–2.14)	0.669
Low-risk HPV	0.87 (0.47–1.61)	0.656	0.85 (0.47–1.55)	0.600	0.47 (0.19–1.16)	0.102
Epithelial cell abnormality on smear	4.28 (2.05–8.92)	<0.001 *	3.73 (1.80–7.72)	<0.001 *	3.50 (1.50–8.16)	0.004 *
Positive colposcopy	3.39 (2.00–5.73)	<0.001 *	3.17 (1.92–5.23)	<0.001 *	3.24 (1.68–6.28)	<0.001 *

OR: odds ratio; CI: confidence interval; * *p* < 0.05. ORs express associations with the odds of the outcome, not relative risks. Biopsy CIN2+ model: Cox and Snell R^2^ = 0.097; Nagelkerke R^2^ = 0.191; Hosmer–Lemeshow χ^2^ = 8.84, *p* = 0.356. Variance inflation factors: age 2.14, menopause 2.13, all other variables ≤ 1.09.

**Table 6:** Cross-classification of colposcopic impression and biopsy histology.

Colposcopic Impression	Biopsy <CIN2	Biopsy CIN2+	Total
Negative	419	24	443
Positive	252	61	313
Total	671	85	756

CIN2+: HSIL/CIN2, HSIL/CIN3, or SCC; <CIN2: chronic cervicitis, squamous metaplasia, or LSIL/CIN1. κ (95% CI) = 0.158 (0.078–0.237). Diagnostic performance measures are presented in Table 7.

**Table 7:** Diagnostic performance of colposcopic impression according to the reference standard.

Reference Standard	Sensitivity, %	Specificity, %	PPV, %	NPV, %	Missed by Colposcopy, %	LR+/LR−
Biopsy CIN2+ (*n* = 85)	71.8 (61.4–80.2); 61/85	62.4 (58.7–66.0); 419/671	19.5 (15.5–24.2); 61/313	94.6 (92.1–96.3); 419/443	28.2 (19.8–38.6); 24/85	1.91 (1.62–2.25)/0.45 (0.32–0.64)
Highest-grade CIN2+ (*n* = 92)	70.7 (60.7–79.0); 65/92	62.7 (58.9–66.2); 416/664	20.8 (16.6–25.6); 65/313	93.9 (91.3–95.8); 416/443	29.3 (21.0–39.3); 27/92	1.89 (1.60–2.23)/0.47 (0.34–0.65)
Biopsy CIN3+ (*n* = 53)	73.6 (60.4–83.6); 39/53	61.0 (57.4–64.6); 429/703	12.5 (9.3–16.6); 39/313	96.8 (94.8–98.1); 429/443	26.4 (16.4–39.6); 14/53	1.89 (1.57–2.27)/0.43 (0.28–0.68)

Values are percentages (95% Wilson confidence intervals) with numerators and denominators. Highest grade: highest histological grade across biopsy and conization specimens. LR: likelihood ratio (with 95% confidence interval, log method); PPV: positive predictive value; NPV: negative predictive value. Missed by colposcopy: proportion of cases with a negative colposcopic impression (1 − sensitivity).

**Table 8:** Concordance between biopsy and conization diagnoses in women who underwent both procedures (*n* = 53).

Biopsy\Conization	Cervicitis	Metaplasia	LSIL (CIN1)	HSIL (CIN2)	HSIL (CIN3)	SCC	Total
Chronic cervicitis	1	0	1	3	4	0	9
Squamous metaplasia	0	2	1	0	0	0	3
LSIL (CIN1)	2	0	2	0	0	0	4
HSIL (CIN2)	1	1	4	6	3	0	15
HSIL (CIN3)	2	2	1	2	14	1	22
Total	6	5	9	11	21	1	53

Rows: biopsy diagnosis; columns: conization diagnosis. Concordant CIN2+/<CIN2 classification: 35/53 (66.0%). Biopsy <CIN2 with conization CIN2+: 7/16 (43.8%); biopsy CIN2+ with conization <CIN2: 11/37 (29.7%).

## Data Availability

The datasets used and analyzed in this study are available from the corresponding author upon reasonable request due to patient privacy restrictions.

## Supplementary Materials

The following supporting information can be downloaded at https://www.mdpi.com/article/10.3390/diagnostics16213685/s1, Table S1: Distribution of certain descriptive and reproductive characteristics according to HPV types; Table S2: Distribution of smear cytology, biopsy, conization, and colposcopy results according to HPV types; Table S3: Distribution of biopsy localization according to HPV types; Table S4: STARD 2015 checklist.

## Abbreviations

The following abbreviations are used in this manuscript:

AGC	atypical glandular cells
ASC-H	atypical squamous cells, cannot exclude HSIL
ASC-US	atypical squamous cells of undetermined significance
ASCCP	American Society for Colposcopy and Cervical Pathology
AUC	area under the receiver operating characteristic curve
CI	confidence interval
CIN	cervical intraepithelial neoplasia
CIN2+	cervical intraepithelial neoplasia grade 2 or worse
FDR	false discovery rate
H&E	hematoxylin and eosin
HPV	human papillomavirus
hrHPV	high-risk human papillomavirus
HSIL	high-grade squamous intraepithelial lesion
IFCPC	International Federation for Cervical Pathology and Colposcopy
IQR	interquartile range
LSIL	low-grade squamous intraepithelial lesion
LR	likelihood ratio
NPV	negative predictive value
OR	odds ratio
PPV	positive predictive value
ROC	receiver operating characteristic
SCC	squamous cell carcinoma
STARD	Standards for Reporting of Diagnostic Accuracy Studies
VIF	variance inflation factor
