# Supplementary material for: Correlation Between Clinicopathological Findings, Cervical Smear, and HPV Results: A Retrospective Study of Women Referred for Colposcopy
Source: Diagnostics (Basel). 2026 Nov 7;16(21):3685. doi: 10.3390/diagnostics16213685 (PMC13763466; doi:10.3390/diagnostics16213685)
Supplement: Supplementary file 1 [file diagnostics-16-03685-s001.zip › diagnostics-4645884-supplementary.pdf]

Supplementary Materials

Correlation between Clinicopathological Findings, Cervical Smear, and HPV Results: A Retrospective Study of Women Referred for Colposcopy

Table S1. Distribution of certain descriptive and reproductive characteristics according to HPV types.

	**HPV-18**			**HPV-16**			**Other high-risk HPV**			**Low-risk HPV**		
	**(–) n = 668**	**(+) n = 88**	**p**	**(–) n = 574**	**(+) n = 182**	**p**	**(–) n = 325**	**(+) n = 431**	**p**	**(–) n = 592**	**(+) n = 164**	**p**
Age (years)	44 (37–51)	44 (39–49)	0.797	45 (38–51)	43 (36–50)	0.210	45 (38–51)	44 (37–51)	0.220	44 (37–51)	44 (36–50)	0.320
Age group			0.228			0.060			0.440			0.669
<40	221 (33.1)	25 (28.4)		174 (30.3)	72 (39.6)		106 (32.6)	140 (32.5)		188 (31.8)	58 (35.4)	
40–49	248 (37.1)	41 (46.6)		229 (39.9)	60 (33.0)		117 (36.0)	172 (39.9)		228 (38.5)	61 (37.2)	
≥50	199 (29.8)	22 (25.0)		171 (29.8)	50 (27.5)		102 (31.4)	119 (27.6)		176 (29.7)	45 (27.4)	
Childbirth status	610 (92.1)	78 (89.7)	0.425	531 (93.3)	157 (87.2)	0.009*	299 (92.3)	389 (91.5)	0.708	542 (92.5)	146 (89.6)	0.228
Mode of delivery			0.034*			0.847			0.089			0.753
Vaginal delivery	334 (54.8)	42 (53.8)		288 (54.2)	88 (56.1)		177 (59.2)	199 (51.2)		295 (54.4)	81 (55.5)	
Cesarean section	209 (34.3)	20 (25.6)		177 (33.3)	52 (33.1)		87 (29.1)	142 (36.5)		179 (33.0)	50 (34.2)	
Both vaginal delivery and cesarean section	67 (11.0)	16 (20.5)		66 (12.4)	17 (10.8)		35 (11.7)	48 (12.3)		68 (12.5)	15 (10.3)	
Menopause	224 (33.5)	23 (26.1)	0.164	191 (33.3)	56 (30.8)	0.530	111 (34.2)	136 (31.6)	0.451	198 (33.4)	49 (29.9)	0.389
Immunosuppression	37 (5.6)	1 (1.1)	0.112	32 (5.6)	6 (3.3)	0.229	23 (7.1)	15 (3.5)	0.027*	26 (4.4)	12 (7.4)	0.127

Continuous variables are presented as median (first quartile–third quartile), and categorical variables as number (column percentage); * $p < 0.05$. Denominators for childbirth status (n = 749) and immunosuppression (n = 753) exclude women with missing data.

Table S2. Distribution of smear cytology, biopsy, conization, and colposcopy results according to HPV types.

	HPV-18			HPV-16			Other high-risk HPV			Low-risk HPV		
	(–) n = 668	(+) n = 88	p	(–) n = 574	(+) n = 182	p	(–) n = 325	(+) n = 431	p	(–) n = 592	(+) n = 164	p
Epithelial cell abnormality on smear	50 (7.5)	2 (2.3)	0.069	35 (6.1)	17 (9.3)	0.132	35 (10.8)	17 (3.9)	<0.001*	41 (6.9)	11 (6.7)	0.922
Type of epithelial cell abnormality			0.968			0.306			0.607			0.614
ASC-US	39 (78.0)	2 (100.0)		30 (85.7)	11 (64.7)		26 (74.3)	15 (88.2)		31 (75.6)	10 (90.9)	
ASC-H	4 (8.0)	0		2 (5.7)	2 (11.8)		4 (11.4)	0		4 (9.8)	0	
AGC	3 (6.0)	0		2 (5.7)	1 (5.9)		2 (5.7)	1 (5.9)		2 (4.9)	1 (9.1)	
LSIL	1 (2.0)	0		0	1 (5.9)		1 (2.9)	0		1 (2.4)	0	
HSIL	3 (6.0)	0		1 (2.9)	2 (11.8)		2 (5.7)	1 (5.9)		3 (7.3)	0	
Inflammation on smear	537 (80.4)	71 (80.7)	0.948	467 (81.4)	141 (77.5)	0.250	258 (79.4)	350 (81.2)	0.532	471 (79.6)	137 (83.5)	0.256
Inflammation findings			0.172			0.072			0.950			0.871
Inflammation on smear	271 (50.5)	40 (56.3)		234 (50.1)	77 (54.6)		133 (51.6)	178 (50.9)		243 (51.6)	68 (49.6)	
Severe inflammation	216 (40.2)	29 (40.8)		198 (42.4)	47 (33.3)		104 (40.3)	141 (40.3)		189 (40.1)	56 (40.9)	
Mild inflammation	50 (9.3)	2 (2.8)		35 (7.5)	17 (12.1)		21 (8.1)	31 (8.9)		39 (8.3)	13 (9.5)	
Atrophy on smear	63 (9.4)	5 (5.7)	0.248	55 (9.6)	13 (7.1)	0.316	28 (8.6)	40 (9.3)	0.752	59 (10.0)	9 (5.5)	0.076
CIN2+ on biopsy	73 (10.9)	12 (13.6)	0.450	43 (7.5)	42 (23.1)	<0.001*	38 (11.7)	47 (10.9)	0.734	69 (11.7)	16 (9.8)	0.496
Cervical biopsy			0.132			<0.001*			0.304			0.142
Chronic cervicitis	478 (71.6)	56 (63.6)		427 (74.4)	107 (58.8)		227 (69.8)	307 (71.2)		412 (69.6)	122 (74.4)	
Squamous metaplasia	76 (11.4)	11 (12.5)		67 (11.7)	20 (11.0)		43 (13.2)	44 (10.2)		68 (11.5)	19 (11.6)	
LSIL (CIN1)	41 (6.1)	9 (10.2)		37 (6.4)	13 (7.1)		17 (5.2)	33 (7.7)		43 (7.3)	7 (4.3)	
HSIL (CIN3)	42 (6.3)	3 (3.4)		17 (3.0)	28 (15.4)		22 (6.8)	23 (5.3)		41 (6.9)	4 (2.4)	
HSIL (CIN2)	25 (3.7)	7 (8.0)		20 (3.5)	12 (6.6)		11 (3.4)	21 (4.9)		22 (3.7)	10 (6.1)	
SCC	6 (0.9)	2 (2.3)		6 (1.0)	2 (1.1)		5 (1.5)	3 (0.7)		6 (1.0)	2 (1.2)	
Conization	47 (7.0)	6 (6.8)	0.940	27 (4.7)	26 (14.3)	<0.001*	24 (7.4)	29 (6.7)	0.727	43 (7.3)	10 (6.1)	0.605
Conization findings			0.012*			0.098			0.441			0.632
HSIL (CIN3)	19 (40.4)	2 (33.3)		6 (22.2)	15 (57.7)		12 (50.0)	9 (31.0)		18 (41.9)	3 (30.0)	
HSIL (CIN2)	11 (23.4)	0		7 (25.9)	4 (15.4)		4 (16.7)	7 (24.1)		8 (18.6)	3 (30.0)	
LSIL (CIN1)	9 (19.1)	0		7 (25.9)	2 (7.7)		2 (8.3)	7 (24.1)		6 (14.0)	3 (30.0)	
Chronic cervicitis	5 (10.6)	1 (16.7)		4 (14.8)	2 (7.7)		3 (12.5)	3 (10.3)		5 (11.6)	1 (10.0)	
Squamous metaplasia	2 (4.3)	3 (50.0)		2 (7.4)	3 (11.5)		3 (12.5)	2 (6.9)		5 (11.6)	0	
SCC	1 (2.1)	0		1 (3.7)	0		0	1 (3.4)		1 (2.3)	0	
Indication for colposcopy			0.001*			<0.001*			<0.001*			<0.001*
HPV	527 (78.9)	87 (98.9)		436 (76.0)	178 (97.8)		200 (61.5)	414 (96.1)		459 (77.5)	155 (94.5)	
Cervical lesion	83 (12.4)	1 (1.1)		83 (14.5)	1 (0.5)		79 (24.3)	5 (1.2)		79 (13.3)	5 (3.0)	
Direct smear result (ASC-US, ASC-H)	18 (2.7)	0		18 (3.1)	0		16 (4.9)	2 (0.5)		16 (2.7)	2 (1.2)	

	HPV-18			HPV-16			Other high-risk HPV			Low-risk HPV		
	**(–) n = 668**	**(+) n = 88**	**p**	**(–) n = 574**	**(+) n = 182**	**p**	**(–) n = 325**	**(+) n = 431**	**p**	**(–) n = 592**	**(+) n = 164**	**p**
HSIL	11 (1.6)	0		8 (1.4)	3 (1.6)		4 (1.2)	7 (1.6)		10 (1.7)	1 (0.6)	
LSIL	10 (1.5)	0		10 (1.7)	0		8 (2.5)	2 (0.5)		10 (1.7)	0	
Other	19 (2.8)	0		19 (3.3)	0		18 (5.5)	1 (0.2)		18 (3.0)	1 (0.6)	
Positive colposcopy	262 (39.2)	51 (58.0)	<0.001*	202 (35.2)	111 (61.0)	<0.001*	142 (43.7)	171 (39.7)	0.267	255 (43.1)	58 (35.4)	0.076
Colposcopy findings (n = 313)												
Acetowhite epithelium	184 (70.2)	40 (78.4)	0.235	144 (71.3)	80 (72.1)	0.883	93 (65.5)	131 (76.6)	0.030*	183 (71.8)	41 (70.7)	0.870
Atypical vascularization	58 (22.1)	15 (29.4)	0.261	48 (23.8)	25 (22.5)	0.804	40 (28.2)	33 (19.3)	0.065	63 (24.7)	10 (17.2)	0.225
Mosaicism	33 (12.6)	4 (7.8)	0.336	21 (10.4)	16 (14.4)	0.292	16 (11.3)	21 (12.3)	0.782	32 (12.5)	5 (8.6)	0.403
Punctation	25 (9.5)	3 (5.9)	0.592	15 (7.4)	13 (11.7)	0.204	14 (9.9)	14 (8.2)	0.606	20 (7.8)	8 (13.8)	0.152
Border-in-border	2 (0.8)	1 (2.0)	0.415	2 (1.0)	1 (0.9)	1.000	1 (0.7)	2 (1.2)	1.000	3 (1.2)	0 (0.0)	1.000

Variables are presented as number (column percentage); * $p < 0.05$. Percentages of colposcopic findings are calculated among women with a positive colposcopy. The comparison of conization findings by HPV-18 status is based on a small number of women and should be interpreted with caution.

Table S3. Distribution of biopsy localization according to HPV types.

	**HPV-18**			**HPV-16**			**Other high-risk HPV**			**Low-risk HPV**		
	**(–) n = 668**	**(+) n = 88**	**p**	**(–) n = 574**	**(+) n = 182**	**p**	**(–) n = 325**	**(+) n = 431**	**p**	**(–) n = 592**	**(+) n = 164**	**p**
Biopsy localization												
1 o'clock position	56 (8.4)	9 (10.2)	0.562	47 (8.2)	18 (9.9)	0.475	28 (8.6)	37 (8.6)	0.988	54 (9.1)	11 (6.7)	0.329
2 o'clock position	22 (3.3)	2 (2.3)	1.000	14 (2.4)	10 (5.5)	0.040*	10 (3.1)	14 (3.2)	0.894	19 (3.2)	5 (3.0)	0.917
3 o'clock position	343 (51.3)	43 (48.9)	0.661	307 (53.5)	79 (43.4)	0.018*	165 (50.8)	221 (51.3)	0.890	304 (51.4)	82 (50.0)	0.759
4 o'clock position	29 (4.3)	2 (2.3)	0.566	23 (4.0)	8 (4.4)	0.818	5 (1.5)	26 (6.0)	0.002*	28 (4.7)	3 (1.8)	0.097
5 o'clock position	45 (6.7)	12 (13.6)	0.021*	38 (6.6)	19 (10.4)	0.089	19 (5.8)	38 (8.8)	0.126	47 (7.9)	10 (6.1)	0.429
6 o'clock position	387 (57.9)	49 (55.7)	0.688	336 (58.5)	100 (54.9)	0.393	192 (59.1)	244 (56.6)	0.497	334 (56.4)	102 (62.2)	0.185
7 o'clock position	56 (8.4)	9 (10.2)	0.562	47 (8.2)	18 (9.9)	0.475	25 (7.7)	40 (9.3)	0.441	55 (9.3)	10 (6.1)	0.197
8 o'clock position	29 (4.3)	4 (4.5)	0.787	27 (4.7)	6 (3.3)	0.418	12 (3.7)	21 (4.9)	0.432	30 (5.1)	3 (1.8)	0.072
9 o'clock position	344 (51.5)	41 (46.6)	0.387	307 (53.5)	78 (42.9)	0.012*	177 (54.5)	208 (48.3)	0.091	289 (48.8)	96 (58.5)	0.028*
10 o'clock position	25 (3.7)	4 (4.5)	0.765	23 (4.0)	6 (3.3)	0.664	14 (4.3)	15 (3.5)	0.558	28 (4.7)	1 (0.6)	0.015*
11 o'clock position	55 (8.2)	11 (12.5)	0.183	48 (8.4)	18 (9.9)	0.525	24 (7.4)	42 (9.7)	0.255	54 (9.1)	12 (7.3)	0.469
12 o'clock position	373 (55.8)	52 (59.1)	0.563	339 (59.1)	86 (47.3)	0.005*	183 (56.3)	242 (56.1)	0.965	323 (54.6)	102 (62.2)	0.081
Quadrant localization												
Q1	409 (61.2)	51 (58.0)	0.554	360 (62.7)	100 (54.9)	0.061	198 (60.9)	262 (60.8)	0.970	364 (61.5)	96 (58.5)	0.493
Q2	442 (66.2)	59 (67.0)	0.870	383 (66.7)	118 (64.8)	0.638	210 (64.6)	291 (67.5)	0.403	389 (65.7)	112 (68.3)	0.536
Q3	412 (61.7)	51 (58.0)	0.501	366 (63.8)	97 (53.3)	0.012*	205 (63.1)	258 (59.9)	0.369	355 (60.0)	108 (65.9)	0.171
Q4	441 (66.0)	62 (70.5)	0.407	394 (68.6)	109 (59.9)	0.029*	213 (65.5)	290 (67.3)	0.614	390 (65.9)	113 (68.9)	0.468
Right (Q1+Q2)	536 (80.2)	72 (81.8)	0.726	464 (80.8)	144 (79.1)	0.611	254 (78.2)	354 (82.1)	0.172	480 (81.1)	128 (78.0)	0.386
Left (Q3+Q4)	536 (80.2)	73 (83.0)	0.545	471 (82.1)	138 (75.8)	0.064	262 (80.6)	347 (80.5)	0.971	474 (80.1)	135 (82.3)	0.520
Top (Q1+Q4)	528 (79.0)	72 (81.8)	0.545	459 (80.0)	141 (77.5)	0.469	252 (77.5)	348 (80.7)	0.281	472 (79.7)	128 (78.0)	0.638
Bottom (Q2+Q3)	534 (79.9)	71 (80.7)	0.870	464 (80.8)	141 (77.5)	0.323	260 (80.0)	345 (80.0)	0.987	469 (79.2)	136 (82.9)	0.294

Variables are presented as number (column percentage). Q1: 1–3 o'clock; Q2: 4–6 o'clock; Q3: 7–9 o'clock; Q4: 10–12 o'clock; * $p < 0.05$ (unadjusted). These analyses were exploratory. After Benjamini–Hochberg false discovery rate correction across the 80 comparisons in this table, none remained significant (smallest q values: HPV-18, 0.242; HPV-16, 0.206; other high-risk HPV, 0.163; low-risk HPV, 0.237). Biopsy clock positions were not recorded in 17 women.

Table S4. STARD 2015 checklist.

Section and topic	No.	Item	Reported in the revised manuscript
Title or abstract	1	Identification as a study of diagnostic accuracy using at least one measure of accuracy	Abstract
Abstract	2	Structured summary of study design, methods, results, and conclusions	Abstract
Introduction	3	Scientific and clinical background, including the intended use and clinical role of the index test	Section 1, paragraphs 3–4
	4	Study objectives and hypotheses	Section 1, final paragraph
Methods: study design	5	Whether data collection was planned before (prospective) or after (retrospective) the index test and reference standard were performed	Section 2.1 (retrospective)
Participants	6	Eligibility criteria	Section 2.1
	7	On what basis potentially eligible participants were identified	Section 2.1 (colposcopy records retrieved from hospital archives)
	8	Where and when potentially eligible participants were identified (setting, location, and dates)	Section 2.1 (tertiary center, Konya, Türkiye; January 2022–June 2025)
	9	Whether participants formed a consecutive, random, or convenience series	Section 2.1 (all eligible women in the study period)
Test methods	10a	Index test, in sufficient detail to allow replication	Sections 2.3 and 2.4
	10b	Reference standard, in sufficient detail to allow replication	Sections 2.4 and 2.5
	11	Rationale for choosing the reference standard (if alternatives exist)	Section 2.5 (biopsy as primary; highest grade across biopsy and conization as secondary)
	12a	Definition of and rationale for test positivity cut-offs or result categories of the index test, distinguishing pre-specified from exploratory	Section 2.4 (any IFCPC abnormal finding = positive)
	12b	Definition of and rationale for test positivity cut-offs or result categories of the reference standard	Section 2.5 (CIN2+ vs. <CIN2; CIN3+)
	13a	Whether clinical information and reference standard results were available to the performers or readers of the index test	Section 2.4 (HPV and cytology available; colposcopic impression recorded before histology)
	13b	Whether clinical information and index test results were available to the assessors of the reference standard	Not documented in the retrospective records; routine practice (Section 4.1)
Analysis	14	Methods for estimating or comparing measures of diagnostic accuracy	Section 2.6
	15	How indeterminate index test or reference standard results were handled	Section 2.1 (nondiagnostic smear and invalid HPV result excluded)
	16	How missing data on the index test and reference standard were handled	Section 2.1, Section 3.1, Table 1 (women without biopsy excluded; available-case denominators reported)
	17	Any analyses of variability in diagnostic accuracy, distinguishing pre-specified from exploratory	Section 2.6, Table 7 (secondary reference standards; exploratory analyses with FDR correction)
	18	Intended sample size and how it was determined	Not applicable (retrospective study including all eligible records)
Results: participants	19	Flow of participants, using a diagram	Figure 3
	20	Baseline demographic and clinical characteristics of participants	Tables 1 and 2
	21a	Distribution of severity of disease in those with the target condition	Table 1 (HSIL/CIN2, HSIL/CIN3, SCC)
	21b	Distribution of alternative diagnoses in those without the target condition	Table 1 (chronic cervicitis, squamous metaplasia, LSIL/CIN1)
	22	Time interval and any clinical interventions between index test and reference standard	Section 2.4: HPV and cytology results available at colposcopy; biopsies obtained during the index colposcopy; conization after biopsy results. Exact intervals and any intervening treatment not systematically documented (Section 4.1)

Section and topic	No.	Item	Reported in the revised manuscript
Test results	23	Cross tabulation of the index test results (or their distribution) by the results of the reference standard	Tables 6 and 8; Figure 3
	24	Estimates of diagnostic accuracy and their precision (such as 95% confidence intervals)	Table 7 (including likelihood ratios with 95% CIs); Section 3.3
	25	Any adverse events from performing the index test or the reference standard	Not recorded (retrospective study)
Discussion	26	Study limitations, including sources of potential bias, statistical uncertainty, and generalizability	Section 4.1
	27	Implications for practice, including the intended use and clinical role of the index test	Sections 4 and 5
Other information	28	Registration number and name of registry	Not registered (retrospective observational study)
	29	Where the full study protocol can be accessed	Approved ethics committee protocol available from the corresponding author on request
	30	Sources of funding and other support; role of funders	Funding statement (no external funding)

STARD: Standards for Reporting of Diagnostic Accuracy Studies (Bossuyt et al., BMJ 2015;351:h5527). Index tests: colposcopic impression (primary), HPV genotyping and cytology (evaluated as predictors). Target condition: CIN2+ on cervical biopsy.
